# Tissue Biomarkers Predicting Lymph Node Status in Cutaneous Melanoma

**DOI:** 10.3390/ijms24010144

**Published:** 2022-12-21

**Authors:** Giulio Rizzetto, Guendalina Lucarini, Edoardo De Simoni, Elisa Molinelli, Monica Mattioli-Belmonte, Annamaria Offidani, Oriana Simonetti

**Affiliations:** 1 Clinic of Dermatology, Department of Clinical and Molecular Sciences, Polytechnic University of Marche, 60126 Ancona, Italy; 2 Histology, Department of Clinical and Molecular Sciences, Polytechnic University of Marche, 60126 Ancona, Italy

**Keywords:** melanoma, lymph nodes, biomarker

## Abstract

Cutaneous melanoma is a severe neoplasm that shows early invasiveness of the lymph nodes draining the primary site, with increased risk of distant metastases and recurrence. The tissue biomarker identification could be a new frontier to predict the risk of early lymph node invasiveness, especially in cases considered by current guidelines to be at low risk of lymph node involvement and not requiring evaluation of the sentinel lymph node (SLN). For this reason, we present a narrative review of the literature, seeking to provide an overview of current tissue biomarkers, particularly vascular endothelium growth factors (VEGF), Tetraspanin CD9, lymphatic vessel endothelial hyaluronan receptor-1 (LYVE-1), D2-40, and gene expression profile test (31-GEP). Among these, 31-GEP seems to be able to provide a distinction between low or high risk for positive SLN classes. VEGF receptor-3 and CD9 expression may be independent predictors of positive SLN. Lastly, LYVE-1 and D2-40 allow an easier assessment of lymph vascular invasion, which can be considered a good predictor of SLN status. In conclusion, biomarkers to assess the lymph node status of cutaneous melanoma patients may play an important role in those cases where the clinician is in doubt whether or not to perform SLN biopsy.

## 1. Introduction

Primary cutaneous melanoma is a severe neoplasm that arises from the degeneration of melanocytes as result of the interaction of genetic, environmental, and acquired factors, which also influence mucosal forms [1,2,3]. The incidence of cutaneous melanoma has been increasing in recent years to become the third most common malignancy [4], with approximately 10–25 cases per 100,000 inhabitants in the European population [5]. The GLOBOCAN estimates of cancer incidence and mortality worldwide in 2020, produced by the International Agency for Research on Cancer, reported that skin melanoma was responsible for 324,635 new cases and 57,043 deaths globally [6]. Melanoma mortality is highest in men older than 65 years and with a lesion thickness greater than 1 mm [7]. For this reason, it is increasingly important to recognize melanoma early in order to reduce the risk of fatal cases [8]. A therapeutic breakthrough in recent years has made it possible to observe increasingly better responses even in patients with advanced disease, thanks to the introduction of targeted therapies and immune checkpoint inhibitors [7,8].

In addition, the melanoma microenvironment, which includes local immune cells, tissue vascular changes, and the local metabolic framework, is important in terms of both disease progression and response to therapy, representing a new field of study to improve the therapeutic approach and increase our understanding of the mechanisms of invasiveness [9,10,11,12]. Indeed, melanoma still represents one of the most aggressive rapid-onset cancers in the world; hence, the study of the molecular mechanisms involved in its spread is crucial, especially considering the possibility of identifying tissue biomarkers capable of predicting the risk of progression [13].

It is known that melanoma tends to result in early invasiveness of the lymph nodes draining the primary site, and the presence of lymph node metastases increases the risk of distant metastases and recurrence [14]. The identification of tissue biomarkers could be a new frontier to predict the risk of early lymph node invasiveness, especially in those cases considered by current guidelines to be at low risk of lymph node involvement and not requiring evaluation of the sentinel lymph node (SLN), currently indicated in cases >1 mm thick. The SLN biopsy should be considered according to a clinician’s judgement in cases of melanoma between 0.8 and 1 mm or <0.8 mm with ulceration or regression greater than 75% [15,16].

The expression of the SLN tissue biomarkers can be tested in the same excised tumor samples, immediately or even simultaneously with the histological diagnosis, further speeding up the patient’s referral to the appropriate therapeutic pathway.

In addition, the identification of reliable biomarkers in predicting lymph node status in melanoma could potentially avoid performing SLN even in patients currently eligible according to guidelines. This could both reduce costs related to melanoma management and avoid overtreatment (Table 1).

For this reason, we perform a narrative review of the literature, seeking to provide an overview of current tissue biomarkers capable of predicting the risk of early lymph node involvement in cutaneous melanoma, and giving our opinion on future perspectives. English language studies were included. A time limit was not used in the choice of articles in order to consider a wider pool and get a complete view. 

## 2. Tissue Biomarkers

### 2.1. Lymphovascular Invasion (LVI)

In the literature, it is reported that lymphovascular invasion (LVI), which consists of the finding during routine histological examination of melanoma tumor cells within lymphatic or hematic vessels, correlates with a worse clinical outcome [17]. The use of highly specific and sensitive antibody biomarkers for melanoma (S-100, Mart-1), for cell proliferation (Ki-67, PCNA), and for lymphatic vessels (LYVE-1, D2-40) allowed a better assessment of LVI. All these biomarkers were assessed by immunohistochemistry directly on primary melanoma tissue. In addition, other parameters that are better analyzed with these biomarkers are lymphatic vessel density (LVD) and peritumoral lymphatic new vessel formation [18,19]. Specifically, LVI was estimated to be present in 50% of LVI-negative patients with hematoxylin/eosin staining after immunohistochemistry (IHC) [20,21,22].

Some studies suggest that intratumoral or peritumoral LVI and LVD may correlate with sentinel node loco-regional lymph node metastasis and patient survival, but the results are not unequivocal [20,23,24,25,26].

### 2.2. VEGF

Lymphangiogenesis is a necessary condition for tumor involvement of the lymphatic and lymph node system. Glycoproteins of the vascular endothelium growth factor (VEGF) family, produced by tumor cells themselves but also tumor-associated fibroblasts and tumor-associated macrophages, help stimulate lymphangiogenesis [27]. VEGF also acts both on endothelial cells and in an autocrine way on tumor cells by promoting their survival and proliferation [28].

The assessment through immunohistochemistry on melanoma tissue of the biomarkers VEGF-C, VEGF-D and the VEGFR receptor to predict SLN positivity has been proposed in the literature, but the results are currently inconsistent. A study by Gallego et al. [29] showed that VEGF-C in the peritumoral versus intratumoral site is associated with SLN positivity. The use of immunohistochemistry and polymerase chain reaction (PCR) for VEGF-C, VEGF-D, and VEGFR-3 proved to be a good predictor of SLN positivity [26,30]. However, other studies have not shown an association among VEGF-A, VEGF-D, or VEGFR receptors [31,32].

Recently, a study by Toberer et al. [33] showed in a statistically significant manner in a sample of 58 patients that VEGFR-3 expression is an independent predictor of SLN positivity and could be used in the future when choosing to perform SLN. VEGF-A was also statistically significantly shown to be more highly expressed in the tumor tissue of SLN-positive patients; however, on binary logistic regression, it did not prove to be an independent factor for SLN outcome.

The ambiguous results require further multicenter prospective studies to better clarify the role of VEGF and VEGFR in predicting SLN involvement.

### 2.3. Tetraspanin CD9

Tetraspanin CD9 is a transmembrane protein that plays a key role in tumor progression, as it functions as a metastasis suppressor in some neoplasms [34]. Specifically, its reduced expression correlates with metastatic progression of the malignancy and poor prognosis. CD9, when overexpressed, enhanced melanocyte motility, suggesting that its overexpression may partly cause the invasion activity of melanoma cells across the Matrigel [35,36].

A recent study [37] conducted on cutaneous melanoma evaluated CD9 expression by immunohistochemistry and immunofluorescence, showing that CD9 was not expressed in thin melanomas, whereas it reappeared in 46% of intermediate and thick melanomas at specific areas of invasion, near or within blood or lymphatic vessels. All of these CD9 stained tumors showed a positive SLN, highlighting that CD9 expression may be a strong predictor of SNB positivity and, therefore, an excellent biomarker for assessing SLN status.

**Figure 1:**
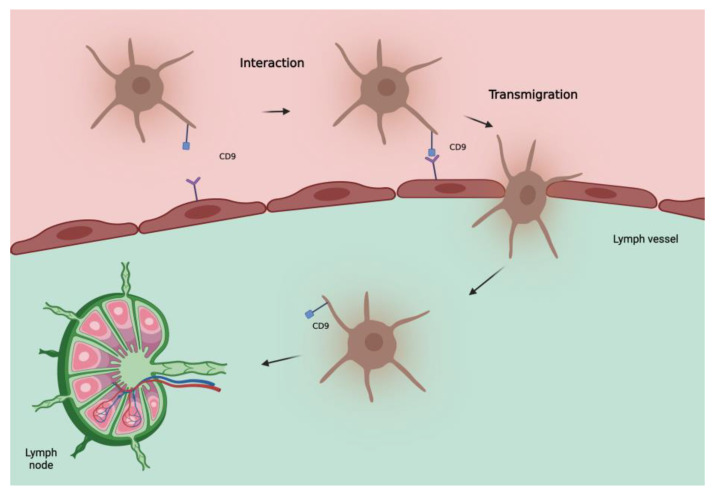
Hypothetical model of involvement of tetraspanin CD9 in metastatic spread of melanoma cells to lymph nodes. CD9, localized at the melanoma cell-endothelial/lymphatic cell contact area during active transmigration of tumor cells across endothelial/lymphatic vessels, facilitates lymph node invasion.

CD9 is also uniformly expressed in melanocytic nevi, leading us to initially consider a protective role of this protein. However, in invasive melanoma, as already seen in other tumors [38,39,40,41,42,43], an increase in CD9 correlates with lymph node metastases, distant metastases, and a worse outcome [37]. This can be explained by considering that tetraspanins can act as both suppressors and promoters of metastases depending on the status of the cell membrane and vesicular structures [44]. Some vitro studies on transendothelial migration of melanoma cells showed a significant role of CD9 in tumor–endothelial/lymphatic cell interaction and vascular dissemination of tumor cells [45]. Therefore, the role of CD9 in determining SLN status seems interesting, as also confirmed by Erovic et al. [46] showing that this tetraspanin is a valid marker for lymphatic endothelial cells and able to promote transmigration of tumor cells through the adherence to lymphatic vessels (Figure 1). Further studies are needed to evaluate its role as a biomarker of the SLN especially in patients with intermediate to thick melanoma.

### 2.4. LYVE-1 and D2-40

Lymphatic vessel endothelial hyaluronan receptor-1 (LYVE-1), a selective marker of lymphatic vessels, and D2-40, an endothelial marker known as podoplanin, were used to assess LVI in cutaneous melanomas. Their distribution was assessed by immunohistochemistry directly on primary melanoma tissue. Using both of these biomarkers, no correlation was found between LVI and SLN positivity [47]. However, if LVI was associated with the presence of intratumoral lymphatic vessels, the possibility of predicting SLN status increased, with a positive predictive value of 80% and a negative predictive value of 72%.

D2-40 was used alone to assess LVI in cutaneous melanoma <2 mm, showing good predictive ability for positive SLN [48]. These data suggest that LVI assessment implemented using LYVE-1 and D2-40 may be an indicator for SLN status.

### 2.5. Gene Expression Profile Test (31-GEP)

31-GEP is a test used to stratify the risk of SLN positivity, identifying high-risk (>5%) patients who are candidates for SLN biopsy. 31-GEP allows evaluation of molecular expression signatures to guide staging in patients with melanoma [49,50,51,52,53,54].

31-GEP is performed on a sample of primary melanoma tissue normally fixed in formalin and embedded in paraffin. Real-time polymerase chain reaction (RT-PCR) is used for the evaluation of expressed genes. From a clinical point of view, this method can be used at the same time as the histological examination. 31-GEP is based on evaluating, directly on the melanoma tissue sample, the expression of gene levels of a panel containing several genes. Specifically, we can find markers for cell migration/chemotaxis/metastasis, secretory molecules, adhesion, lymphocyte invasion, transcription factors, differentiation/proliferation structural proteins, and surface receptors (CXCL14, SPP1, CLCA2, S100A9, S100A8, BAP-1, MGP, GJA1, DSC1, PPL, LTA4H, TRIM29, KRT6B, KRT14, CRABP2, SPRRIB, TACSTD2, CLCA2, ROBO1, CST6, SAP130, ID2, EIF1B, ARG1, AQP1, RBM23, and TYRP1) [55]. 

Genetic tests can identify a class 1 low risk of SLN positivity or a class 2 high risk. Class 1 is considered to have a very low risk of both metastasis and mortality, also providing greater tranquillity for patients and improving their quality of life [53].

DecisionDx-Melanoma has recently been proposed as an additional risk assessment parameter for SLN positivity, integrating the 31-GEP test with other clinical/pathological aspects for the evaluation of SLN in patients with thin melanoma [56,57].

This could, in the near future, introduce a new variable to be considered, highlighting population groups with a >5% risk of SLN positivity, optimizing the treatment.

All the melanoma tissue biomarkers are summarized in Table 2.

## 3. Discussion

In our review, we did not consider biomarkers that did not provide evidence of utility in predicting SLN status from their use on primary tumour tissue. For example, Sry-related box (Sox) 10 is a gene involved in neural crest-derived cell development that regulates the expression of genes involved in melanin production including microphthalmia-associated transcription factor (MiTF) [58,59]. Although this biomarker is used as an aid in the differential diagnosis of melanoma [60], one study also reported its application in the assessment of SLN positivity, but it had no predictive ability at the skin level [61]. Manninen et al. [62] demonstrated that BRAF immunohistochemistry could serve as a useful addition to Breslow thickness and mitotic count for selecting intermediate thickness melanoma patients for SLN biopsy since positive BRAF V600E immunoreactivity correlated with sentinel node involvement (*p* = 0.013) in the entire cohort. In melanomas, the oncogene BRAF is the most commonly mutated (more than 50% of tumors), and 90% of all activating BRAF mutations involve V600E substitution [63]. However, although the result is interesting, this study only enrolled patients with intermediate thickness melanoma, and further studies should include thin and thick melanoma.

We also did not include some descriptive parameters such as microsatellites, thickness, or number of mitoses as they cannot be considered biomarkers in the strict sense. However, we report that, in a systematic review, a significant increase in the frequency of positive SLNs was found in melanomas with a thickness >0.75 mm, presence of microsatellites, and >1 mitosis/mm^2^, highlighting that these are currently the parameters that can guide the clinician in choosing to perform SLN [64]. The statistical limitation reported by the study was a lack of a significant adjusted odds ratio for these parameters. Further studies with larger samples will, therefore, be useful to confirm these hypotheses. In addition, other studies from the Sentinel Lymph Node Group in Melanoma confirmed that, in melanomas, <1 mm SLN positivity is the most important prognostic factor, showing that overall survival (OS) increased with statistical significance in patients who underwent SLN compared to those who did not. For this reason, SLN is now also indicated in thin melanoma cases with a risk of positive SLN greater than 5% [65,66].

VEGF and VEGFR are biomarkers with excellent potential to predict the lymph node status of thin melanoma patients; however, definitive data are lacking. VEGFR-3 in particular has been shown to be an independent predictor of SLN positivity [33] and plays a key role in lymphangiogenesis and lymph node metastasis [67,68].

VEGF and VEGFR-3 appear to be correlated; however, as VEGF can induce receptor expression by an autocrine mechanism [66]. In another study by Lucarini et al. [9] a correlation between VEGF and melanoma thickness was reported, but it was not indicative of lymph node or distant metastasis. The role of a VEGF inhibitor, semaphorin (SEMA) 3A, was also introduced, which is significantly reduced in intermediate and thick melanomas associated with metastasis. In our opinion, the evaluation of VEGFR-3 could be a useful element to discriminate those lesions at high risk of SLN positivity. The role of VEGF and SEMA3A still needs to be studied with larger samples in order to understand whether their correlation could also be useful in predicting SLN status.

CD9 could also be an important marker for SLN status, with CD9 having an increased expression in intermediate and thick melanomas, with an evident increased risk of lymph node metastasis. However, further studies are needed to support this finding. We believe that it may be an interesting biomarker, especially considering its possible increased expression in sites of lymphovascular invasion.

LYVE-1 and D2-40 are already known biomarkers that play an important role in facilitating evidence of melanoma cancer cells within vascular or lymphatic structures, also known as LVI. Although LVI has not been correlated with survival of melanoma patients, a number of studies have highlighted it as an independent factor predicting SLN status [69,70,71,72,73,74,75]. We believe that LVI may be a useful parameter to evaluate in all thin melanoma cases where a clinician is in doubt whether to perform SLN, as suggested by the guidelines [15].

Furthermore, genetic tests, such as 31-GEP, are likely to represent the future for the evaluation of performing SLN, allowing the detection of SLN-positive cases above 5% risk, especially in cases where the clinician is in doubt. The subjective measurements of tumor characteristics or SLN tumor burden can result in discrepancies in diagnosis and under- or overestimation of metastatic risk, leading to under or overtreatment of the patient [76]. As Dillon et al. recently reported [77], the 31-GEP test is a molecular prognostic measure of metastatic risk that can help clinicians to develop appropriate management strategies for the patients with cutaneous melanoma. They demonstrated the clinical utility of the 31-GEP by evaluating the risk appropriate changes in management after receiving 31-GEP results in a large cohort of patients (n = 509): the 31-GEP test led to management plan changes for 51% of patients. Currently, this method is not yet routinely used, but it can be a potential new parameter in clinical practice.

Lastly, extracellular vesicles from melanoma (MEVs) have recently been reported in the literature as promoters of both lymph node invasiveness and then distant metastases [78]. MEVs are membrane-covered vesicles that act as subcellular mediators, capable of carrying a nucleic and proteomic cargo that can perform various immune regulatory functions involved in tumoral diffusion [79]. MEVs seem able to favor the formation of a pre-metastatic niche (PMN), which later allows metastatic lymph node invasion, interacting both with medullary sinus macrophages CD169^+^ and with lymphatic endothelial cells (LECs) [78].

However, the role of MEVs has not yet been evaluated in patients with melanoma as a predictor of early lymph node involvement, although there are interesting potential applications to be developed. In our opinion, the evaluation for specific MEVs from the patient’s serum could not only potentially be a predictor of melanoma screening, as already suggested [80,81], but also show potential effectiveness in identifying the SLN metastatic involvement. Further studies are needed to qualitatively and quantitatively assess the specific MEVs cargoes that can be successfully correlated with SLN positivity.

## 4. Conclusions

Biomarkers to assess the lymph node status of cutaneous melanoma patients may play an important role in those cases where a clinician is in doubt whether or not to perform SLN biopsy. Among these, 31-GEP seems to provide a negative predictive value of 99%, with a distinction into low- and high-risk classes, although it has not yet shown high applicability. VEGF and VEGFR are biomarkers whose roles have yet to be clarified, although it has been shown that VEGFR-3 expression may be an independent predictor of positive SLN. The fact that CD9 expression was found only in melanomas with lymph node metastases highlights that CD9 could be a promising biomarker for predicting SLN positivity, although support by further studies is needed.

Lastly, LYVE-1 and D2-40 allow an easier assessment of LVI, which in turn can be considered a good predictor of SLN status.

## Figures and Tables

**Table 1:** Clinical utility of melanoma tissue biomarkers.

Early diagnosis of lymph node metastasis Identification of the patients for SLN biopsy to avoid invasive procedures Avoiding surgical procedures to reduce hospital costs Providing prognosis and selecting appropriate treatment (adjuvant therapy)

**Table 2:** summary of reviewed biomarkers for SLN positivity.

Biomarker	Rationale	Sample	Results
VEGFR-3 Toberer et al. [33]	Involved in the stimulation of lymphangiogenesis	58 patients	- independent predictor of SLN positivity (ss) - VEGF-A highly expressed in the tumour tissue of SLN-positive patient (ss) but not an independent predictor of SLN positivity
Tetraspanin CD9 Lucarini et al. [37]	Transmembrane protein, key role in tumor progression Both suppressor and promoter of metastases, depending on the status of the cell membrane and vesicular structures	140 patients Melanocytic nevus 20 Primary melanoma 120 - <1 mm (thin) 24 - 1.1–4 mm (intermediate) 56 - >4 mm (thick) 40	- CD9 not expressed in thin melanomas - CD9 reappears in 46% of intermediate and thick melanomas (near or within blood or lymphatic vessels); all of these melanomas showed a positive SLN - CD9 may be an excellent biomarker for assessing SLN status
LYVE-1 Doeden et al. [47]	LYVE-1 selective marker of lymphatic vessels	94 patients	- if LVI associated with intratumoral lymphatic vessels, increased SLN status prediction (positive predictive value 80%, negative predictive value 72%) [46]
D2-40 Fohn et al. [48]	D2-40 endothelial marker (podoplanin) In combination, better histological definition of LVI	158 patients Primary melanomas ≤ 2.0 mm	- D2-40 alone to assess LVI in ≤2 mm melanoma, good positive predictive ability (85.7%) for positive SLN [47]
31-GEP Vetto et al. [55]	Gene expression profile test, (markers for cell migration/chemotaxis/metastasis, secretory molecules, adhesion, lymphocyte invasion, transcription factors, differentiation/proliferation structural proteins and surface receptors) Identifies high-risk (>5%) patients, candidates for SLN	690 patients (total validation cohort, retrospective) staged I–III follow-up median 7 years 1421 patients (prospectively-tested)	- new class identification: class 1, low risk of SLN positivity - class 2, high risk (>5%) of SLN positivity - possibility of avoiding SLN in low-risk patients, reducing costs and overtreatment - validated method proposed by DecisionDx-Melanoma [56]

Vascular endothelium growth factor receptor-3 (VEGF), statistically significant (ss), lymphatic vessel endothelial hyaluronan receptor-1 (LYVE-1), lymphovascular invasion (LVI), sentinel lymph node (SLN), and gene expression profile test (31-GEP).

## Data Availability

Not applicable.

## Abbreviations

GEP	Gene expression profile
LVD	Lymphatic vessel density
LVI	Lymph vascular invasion
LYVE 1	Lymphatic vessel endothelial hyaluronan receptor-1
MEV	Melanoma extracellular vesicle
SLN	Sentinel lymph node
